# Programmed cell death-ligand 1 expression in stromal immune cells is a marker of breast cancer outcome

**DOI:** 10.7150/jca.50441

**Published:** 2020-10-18

**Authors:** Mineui Hong, Jeong Won Kim, Min kyoon Kim, Bong wha Chung, Soo kyung Ahn

**Affiliations:** 1 Department of Pathology, Kangnam Sacred Heart Hospital, Hallym University College of Medicine, 1 Shingil-ro, Youngdeungpo-ku, Seoul, 07441, Korea; 2 Department of Surgery, College of Medicine, Chung-Ang University, 84 Heukseok-ro, Dongjak-gu, Seoul, 06974, Korea; 3 Department of Surgery, Kangnam Sacred Heart Hospital, Hallym University College of Medicine, 1 Shingil-ro, Youngdeungpo-ku, Seoul, 07441, Korea

**Keywords:** PD-L1, breast cancer, prognosis, immune-oncology, stromal immune cells

## Abstract

**Purpose:** The programmed cell death 1 (PD-1)/programmed cell death ligand 1 (PD-L1) axis plays an important role in antitumor immune responses. However, there is considerable inconsistency regarding the prognostic value of PD-L1 expression status in breast cancer. We sought to evaluate the differential prognostic impacts of tumoral versus stromal immune cell PD-L1 expression in primary breast cancer.

**Materials & Methods:** Both tumoral and stromal immune PD-L1 expression in formalin-fixed, paraffin-embedded tumor samples from 233 breast cancer patients without initial stage IV metastases were evaluated by immunohistochemistry using a mouse monoclonal anti-PDL1 antibody. Clinicopathological variables were also documented. A Cox regression model was used to assess the association of tumoral/stromal immune PD-L1 expression with clinical outcome using disease-free survival (DFS) as the primary end point.

**Results:** Both tumoral and stromal immune PD-L1 expression were associated with aggressive tumor characteristics, including higher histologic grade, as well as negative estrogen receptor, negative progesterone receptor, and positive human epithelial growth factor receptor 2 (HER2) status Multivariate analyses further demonstrated that stromal immune cell, but not tumoral, PD-L1 expression was a favorable prognostic factor for survival.

**Conclusions:** Despite its association with aggressive tumor features, PD-L1 expression on stromal immune cells emerged as a positive prognostic biomarker in breast cancer. This pro-survival effect might reflect the presence of a strong antitumor immune response that leads to PD-L1 expression.

## Introduction

Numerous recent studies have focused on the host immune system and its relationship with tumor progression in a variety of solid tumors, including breast cancer. One of the most important immune pathways is the programmed cell death 1 (PD-1)/programmed cell death ligand 1 (PD-L1) pathway.

PD-1, which belongs to the B7-CD28 superfamily, is involved in T cell regulation, and functions as a negative regulator of the immune system, resulting in reduced proliferation of activated CD8^+^ T cells. PD-L1 is expressed in some tumor cells and in activated B cells, T cells, dendritic cells, macrophages, and fibroblastic cells.^1^ PD-L1 binding to PD-1 attenuates the cellular immune response by inducing T-cell apoptosis or exhaustion. PD-L1 plays an important role in tumor immune escape by facilitating PD-1/PD-L1 pathway activation.^2^

It has been shown that PD-L1 is expressed in several malignancies, including breast cancer.^3^ In addition, it has been suggested that higher PD-L1 expression in tumor cell membrane versus stromal immune cells is associated with different clinicopathological features and clinical outcomes in multiple different tumor types. Although PD-L1 expression can be evaluated in both tumor cells and stromal immune cells in breast cancer, the implications of differences in expression between these two compartments remain unclear.

Here, we sought to evaluate difference in PD-L1 expression between tumor cells and stromal immune cells and assess differential prognostic impacts of PD-L1 expression in tumor cells versus stromal immune cells in breast cancer patients.

## Materials & Methods

### Case selection

Tumor samples were collected from 233 female patients with stage I, II, or III breast cancer between 2013 and 2018 at Kangnam Sacred Heart Hospital, Hallym University, Seoul, Korea. Patients that satisfied the following selection criteria were included: (1) those who underwent primary resection; (2) those with no prior treatment; and (3) those with available, complete medical records, including pathologic slides and paraffin blocks of resected specimens. Diagnosis and histological differentiation were performed according to World Health Organization classifications. Staging was based on the American Joint Committee on Cancer staging system (eighth edition).

Tumor samples were assessed using tissue microarrays (TMAs) and immunohistochemistry. Baseline clinicopathological characteristics, factors in patients with metastatic disease, and clinical follow-up data were retrospectively collected from our database. Institutional Review Board approval was obtained (HKS 2018-10-011).

### TMA construction

For TMA construction, all hematoxylin and eosin (H&E)-stained slides were reviewed, and representative areas were carefully selected. Each paraffin-embedded block relevant to H&E-stained slides was punched out using a TMA manufacturing tool (Quick-Ray; Unitma, Seoul, South Korea) and placed into a recipient paraffin block. Three separate tissue cores (2 mm in diameter) were obtained from each tumor specimen.

### Immunohistochemistry

Immunohistochemical detection of PD-L1 and CD8, the latter of which is a marker of tumor-infiltrating lymphocytes (TILs), was performed on 4-μm-thick whole-tissue and TMA sections using an automated immunostainer, according to the manufacturer's protocol (BenchMark XT; Ventana Medical Systems, Tucson, AZ, USA). Appropriate positive and negative controls were included in each run, and all immunohistochemically stained slides were interpreted by a single investigator (ME Hong). Signals were visualized using an Optiview DAB IHC detection kit and Optiview Amplification kit (Ventana).

The primary antibody used for detection of PD-L1 was SP263 (clone SP263; Ventana, Cat. No. 7414905). PD-L1 expression on the cell membrane of tumor cells, with/without cytoplasmic staining, was evaluated. The proportion of PD-L1-positive cells was estimated as a percentage of total tumor cells. PD-L1 staining intensity and percentage of positive tumor cells were scored as follows: 0, no staining or any staining in less than 1% of cells; 1+, weak staining in 1-10% of tumor cells; 2+, moderate staining in 10-50% of tumor cells; and 3+, strong staining in more than 50% of tumor cells.

The primary antibody used for detection of CD8 was SP57 (Ventana, Cat. No. 7904460). The average number of CD8^+^ TILs that also expressed PD-L1 was evaluated semi-quantitatively in TMA fields at 200× magnification. The proportion of CD8^+^ TILs in the tumor and surrounding stroma was evaluated. PD-L1 staining in CD8^+^ TILs was scored as follows: 0, no staining or any staining in less than 1% of cells; 1+, staining in 1-5% of cells; 2+, staining in 5-10% of cells; 3+, staining in 10-25% of cells; 4+, staining in 25-50% of cells; and 5+, staining in more than 50% of cells. Ultimately, staining results were categorized into two groups: TILs-PDL1-low (range, 0% to 10%) and TILs-PDL1-high (range, 11% to 100%).

### Subtyping of breast cancer

Samples were classified into the following four intrinsic breast cancer subtypes based on immunohistochemical findings: (1) luminal A (LumA), (2) luminal B (LumB), (3) HER-2-enriched (HER-2^+^), and (4) basal subtypes. LumA cancers are ER^+^PR^+^HER-2^-^Ki-67^low^; LumB cancers are ER^+^PR^+^HER-2^-^Ki-67^high^ or ER^+^PR^+^HER-2^+^; HER-2-enriched cancers are ER^-^PR^-^HER-2^+^; and basal cancers are typically ER^-^PR^-^HER-2^-^ (triple-negative breast cancers with cytokeratin 5/6 or epidermal growth factor receptor expression) 4.

### Statistical analysis

Associations of PD-L1 expression in tumor and stromal immune cells with categorical variables were tested by chi-square test. Survival was analyzed using Kaplan-Meier survival curves and log-rank tests, and multivariate analyses of survival were performed using Cox regression analysis. Disease-free survival (DFS) was measured from the date of surgery to the date of local recurrence or distant metastasis. Statistical analyses were carried out using SPSS software (version 24; IBM, Armonk, NY, USA), and a *P*-value < 0.05 was considered statistically significant.

## Results

Tumors from 233 patients for which tumor samples and adequate clinical data were available for evaluation of PD-L1 expression in both tumor cells and stromal immune cells were assessed. The age of patients at the time of diagnosis ranged from 23 to 82 years (median, 52.5 years). The median follow-up was 45 months (1-82 months). Among the 233 patients, all of which were free of systemic metastasis at initial presentation, 15 (6.4%) developed recurrence. Patients and tumor characteristics are described in Table 1.

### Association between tumoral PD-L1 expression and clinicopathologic factors

**Fig 1:**
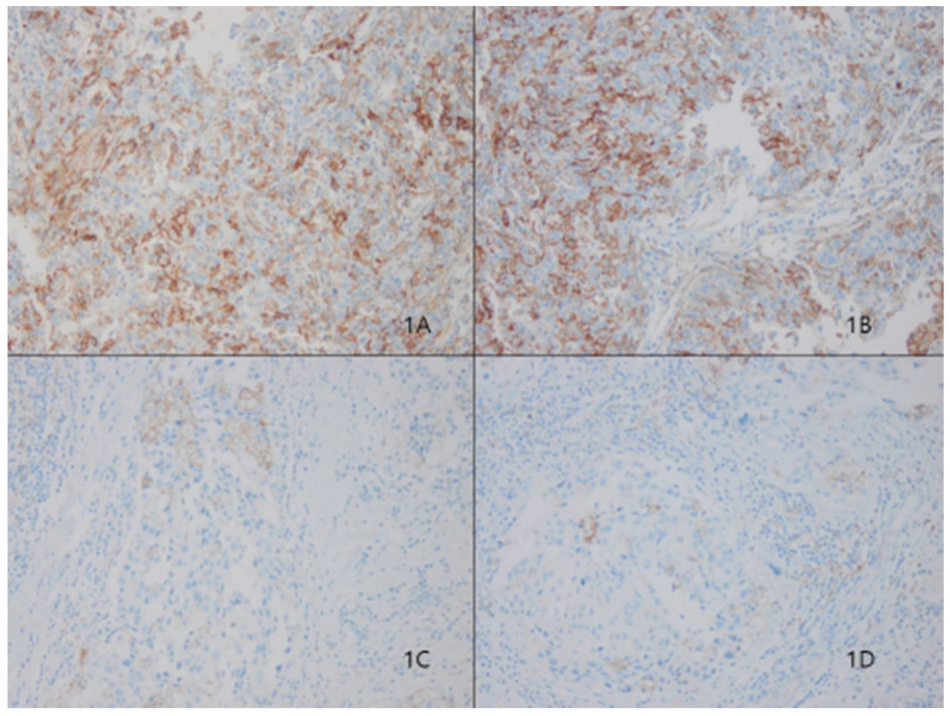
Representative microphotographs of sections from breast cancer samples are showing different tumoral PD-L1 expression compartments. Programmed death ligand 1 (PD-L1) protein 3+ of sp263 (Fig. 1A and 1B), 1+ of sp263 (Fig. 1C and 1D). (200×)

In our cohort, 28 (12%) of 233 patients displayed tumoral PD-L1 expression (Fig. 1). We found a correlation between higher PD-L1 expression and unfavorable classic prognostic factors, including higher histologic grade (HG) (*p*=0.001); negative estrogen receptor (ER) (*p*<0.001), negative progesterone receptor (PR) (*p*=0.007) and positive human epithelial growth factor receptor 2 (HER2) (*p*=0.01) status; and higher Ki67 index (*p*=0.004). We also observed a significant direct association between PD-L1 expression and all evaluated basal cell markers, including cytokeratin 5/6 (*p*<0.001), epithelial growth factor receptor (EGFR) (*p*=0.001), and c-kit (*p*=0.002).

### Association between PD-L1 expression in stromal immune cells and clinicopathologic factors

**Fig 2:**
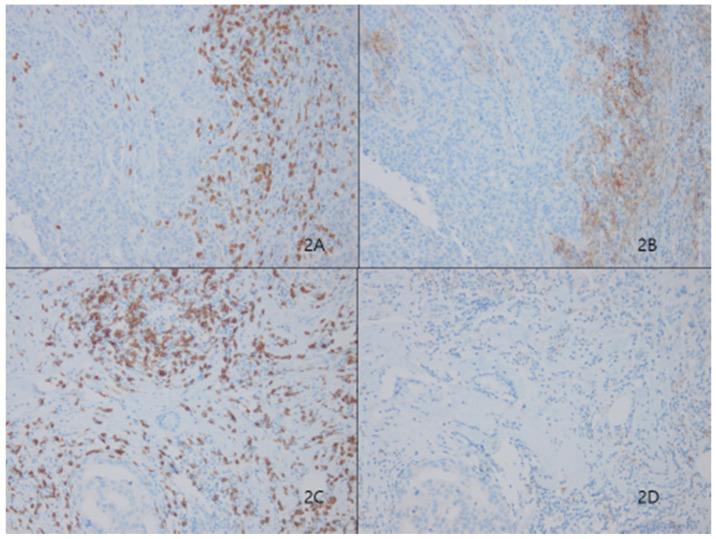
Stromal tumor lymphocytic infiltrates (TILs) evaluated by CD8 (2A) presented significantly higher expression levels of Programmed death ligand 1 (PD-L1, SP263) in Figure 2B. Representative figures of lower expression levels of PD-L1 (Fig. 2D) in matched CD8-positive TILS (Fig. 2C)

Among 233 patients, 66 (28.3%) showed high TILs-PD-L1 expression on stromal immune cells (Fig. 2). Similar to the case for tumoral PD-L1 expression, higher stromal PD-L1 expression was correlated with classic unfavorable prognostic factors, including higher histologic grade (*p*<0.001), negative ER (*p*<0.001), negative PR (*p*<0.001) and positive HER2 (*p*<0.001) status, and higher Ki67 index (*p*<0.001). Stromal PD-L1 expression was also significantly correlated with the basal cell markers, cytokeratin 5/6 (*p*<0.001) and EGFR (*p*=0.001), but not c-kit (*p*=0.085) (Table 1).

### Association between PD-L1 expression and disease-free survival

**Fig 3:**
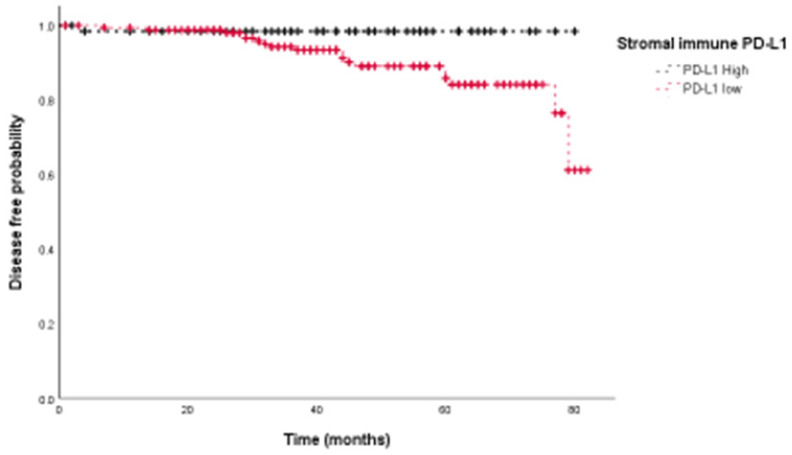
Stromal immune expression of PD-L1 is associated with better disease-free survival in breast cancer patients.

We then assessed the prognostic value of PD-L1 expression in terms of disease free survival (DFS). Univariate analyses showed that PD-L1 expression on stromal immune cells was associated with improved prognosis (HR=0.146, *p*=0.044) (Fig. 3), whereas tumoral PD-L1 expression was not (*p*=0.325). Univariate survival analyses further showed that the presence of lymphovascular invasion (LVI) (p=0.002), negative PR status (p=0.013), and higher HG (*p*=0.027) were significantly associated with poor DFS. Adjuvant therapy including chemotherapy (HR 1.160, 95%CI: 0.266-5.065, p=0.844), radiation therapy (HR 0.421, 95%CI:0.158-1.124, p=0.084) and hormone therapy (HR 0.431, 95%CI:0.175-1.065, p=0.068) did not show significant association with DFS. Multivariate survival analysis showed that PD-L1 expression on stromal immune cells (HR=0.084, p=0.017), the presence of LVI (HR=4.574, p=0.018), and higher HG (HR=3.327, *p*<0.001) were independent prognostic factors for DFS (Table 2).

Finally, we performed the same analyses for each molecular subtype separately. In the basal breast cancer subtype, PD-L1 expression on stromal immune cells showed a trend toward improved prognosis (HR=0.074, 95% CI=0.004-1.234, *p*=0.07). By contrast, no significant influence of PD-L1 expression was seen in luminal A (*p*=0.995), luminal B (*p*=0.972), or HER2-enriched (*p*=0.731) subtypes.

## Discussion

We demonstrated that stromal immune cell, but not tumoral, PD-L1 expression is associated with a favorable prognostic outcome, measured as DFS, in breast cancer patients.

PD-L1 expression in the tumor epithelium was less frequent than that in stromal immune cells, and both tumoral and stromal immune cell PD-L1 expression was predominantly associated with features of aggressive tumor biology, including higher HG as well as negative ER, negative PR, and positive HER2 status. A meta-analysis by Zhang et al 5 also showed that PD-L1 expression is associated with positive lymph node metastasis, higher HG, ER-negativity and triple-negative breast cancer (TNBC) subtype. A possible explanation for this observation is that higher expression of PD-L1 may reflect activation of the PD1/PD-L1 immune checkpoint pathway, leading to immune evasion processes and, ultimately, increased tumor aggressiveness.^6^

Interestingly, despite the association of positive stromal immune PD-L1 expression with aggressive tumor factors, patients with this expression profile showed improved DFS. The relationship between PD-L1 expression and prognosis remains unclear in breast cancer patients. Some studies have reported that positive PD-L1 status is associated with significantly improved overall survival 7, 8, but other studies have not confirmed this finding.^9^-^11^ It has also been reported that increased PD-L1 expression is a favorable prognostic factor in patients with non-small-cell lung cancer (NSCLC) 12, small-cell lung cancer 13, gastric cancer 14, pancreatic cancer 15 and tonsillar cancer.^16^ It has been speculated that, while PD-L1 mediates immune invasion^2^ and would thus be expected to show an association with poor prognosis, expression of PD‑L1 by stromal immune cells may be ineffective in suppressing the immune response and may merely reflect infiltration by lymphocytes, which are associated with a generally good outcome in several malignancies, including breast cancer.^17^ Several studies have described prominent immune cell infiltration, such that tumor-infiltrating lymphocytes (TILs) are associated with PD-L1 expression in breast cancer.^18^ It is possible that PD-L1 expression is associated with a TIL-mediated antitumor inflammatory response rather than tumor immune evasion.

While tumoral membranous expression of PD-L1 is often used as the criterion for PD-L1 positivity in cancers, such as NSCLC,^19^ renal cell carcinoma^20^ and melanoma,^21^ we found no association between tumoral PD-L1 expression and outcome. In contrast, Thompson et al.^22^ reported that both tumoral and stromal PD-L1 expression were associated with worse outcomes in patients with locally advanced stomach cancer. The contrasting reports of the impact of tumoral and stromal PD-L1 expression on survival between our breast cancer patients and the gastric cancer cohort of Thompson et al. suggest that interactions between tumors and tumor-associated stroma might differ among various cancer types. Stromal immune cell expression of PD-L1 in breast cancer has not been well documented in other studies. To date, the precise mechanism by which PD-L1 expression on stromal cells exerts antitumoral effects within the tumor microenvironment remains elusive.^23^

In addition, a consideration of specific breast cancer subtype suggests that PD‑L1 expression may retain a positive prognostic role only in the basal subtype. Loi et al. 24 commented on the consistency of positive associations between TILs in primary TNBC and prognosis in three clinical trials, concluding that immunity is important for the outcome of primary TNBC.

Limitations of our study include the small number of patients and its retrospective, single-center design. There is no established consensus regarding PD-L1 scoring, and considerable heterogeneity exists in PD-L1 antibodies used for immunohistochemical staining across studies. Strengths include that this is one of the first studies to describe the importance of stromal PD-L1 expression in favorable prognostic outcomes in breast cancer patients. Future large-scale studies will be necessary to evaluate the biological and clinical implications of differential expression of PD-L1 by tumor cells and stromal immune cells.

In conclusion, our results indicate that PD-L1 expression on stromal immune cells is associated with favorable prognostic outcome, specifically DFS, in breast cancer. PD-L1 expression was significantly associated with a series of unfavorable clinicopathological parameters, including higher HG, higher Ki67, ER and PR negativity and positive HER2 status. This information may be helpful to clinicians attempting to screen candidates for anti PD-1/PD-L1 therapy.

## Figures and Tables

**Table 1:** Clinical and pathological characteristics and tumoral and stromal immune PD-L1 expression

	Tumoral PDL1			Immune stromal PDL1		
	Positive (≥1%) (n=28)	Negative (<1%) (n=205)	*p*-value	High (>10%) (n=66)	Low (≤10%) (n=167)	*p*-value
Age			0.424			0.181
≤50 yr	11(39.3%)	97(47.3%)		26(39.4%)	82(49.1%)	
>50 yr	17(60.7%)	108(52.7%)		40(60.6%)	85(50.9%)	
Menopause			0.438			0.903
Premenopause	12(50%)	79(41.4%)		25(41.7%)	66(42.6%)	
Postmenopause	12(50%)	112(58.6%)		35(58.3%)	89(57.4%)	
Multiplicity			0.105			0.302
Yes	4(14.3%)	49(28.8%)		21(31.8%)	42(25.1%)	
No	24(85.7%)	146(71.2%)		45(68.2%)	125(74.9%)	
Histology			0.004			0.923
IDC	22(78.6%	192(93.8%)		56(84.8%)	158(94.6%)	
ILC	0(0%)	2(1.0%)		1(1.5%)	1(0.6%)	
Medullary ca	5(17.9%)	1(0.5%)		6(9.1%)	0(0%)	
Mucinous ca	1(3.6%)	5(2.4%)		1(1.5%)	5(3%)	
Tubular	0(0%)	3(1.5%)		1(1.5%)	2(1.2%)	
metaplastic	0(0%)	3(1%)		1(1.5%)	1(0.6%)	
Tumor size			0.534			0.428
T1&T2	13(46.4%)	108(52.7%)		37(56.1%)	84(50.3%)	
T3&T4	15(53.6%)	97(47.3%)		29(43.9%)	83(49.7%)	
LN meta			0.330			0.189
Negative	20(71.4%)	127(62%)		46(69.7%)	101(60.5%)	
Positive	8(28.6%)	78(38%)		20(30.3%)	66(39.5%)	
Stage			0.430			0.579
Stage1	13(46.4%)	84(41%)		31(47.05)	66(39.5%)	
Stage 2	13(46.4%)	87(42.4%)		26(39.4%)	74(44.3%)	
Stage 3	2(7.1%)	34(16.6%)		9(13.6%)	27(16.2%)	
HG			0.001			<0.001
1&2	9(33.3%)	132(66%)		21(32.8%)	120(73.6%)	
3	18(66.7%)	68(34%)		43(67.2%)	43(26.4%)	
LVI			0.776			0.661
Positive	8(28.6%)	64(31.2%)		19(28.8%)	53(31.7%)	
Negative	20(71.4%)	141(68.8%)		47(71.2%)	114(68.3%)	
ER			<0.001			<0.001
Positive	12(42.9%)	160(78.0%)		33(50.0%)	139(83.2%)	
Negative	16(57.1%)	45(22.0%)		33(50.0%)	28(16.8%)	
PR			0.007			<0.001
Positive	12(42.9%)	141(68.8%)		30(45.5%)	123(73.7%)	
Negative	16(57.1%)	64(31.2%)		36(54.5%)	44(26.3%)	
HER2			0.01			<0.001
Positive	13(46.4%)	46(23.7%)		30(46.2%)	29(18.5%)	
Negative	15(53.6%)	148(76.3%)		35(53.8%)	128(81.5%)	
Ki67			0.004			<0.001
≤14%	4(14.3%)	91(44.4%)		12(18.8%)	83(49.7%)	
>14%	23(82.1%)	113(55.1%)		52(81.3%)	84(50.3%)	
EGFR			0.035			0.001
Positive	6(23.1%)	19(9.4%)		14(22.2%)	11(6.6%)	
Negative	20(76.9%)	184(90.6%)		49(77.8%)	155(93.4%)	
Cytokeratin 5/6			<0.001			<0.001
Positive	12(44.4%)	21(10.6%)		20(31.7%)	13(8%)	
Negative	15(55.6%)	177(88.9%)		43(68.3%)	149(92%)	
C-kit			0.002			0.085
Positive	10(38.5%)	28(14.4%)		15(24.2%)	23(14.5%)	
Negative	16(61.5%)	167(85.6%)		47(75.8%)	136(85.5%)	
Subtype			0.009			<0.001
Luminal A	3(10.7%)	68(35.1%)		5(7.7%)	66(42.0%)	
Luminal B	11(39.3%)	82(42.3%)		29(44.6%)	64(40.8%)	
Basal	9(32.1%)	27(13.9%)		19(29.2%)	17(10.8%)	
HER2	5(17.9%)	17(8.8%)		12(18.5%)	10(6.4%)	
Radiation Therapy			0.686			0.924
Yes	20(76.9%)	159(80.3%)		49(80.3%)	130(79.8%)	
No	6(23.1%)	39(19.7%0		12(19.7%0	33(20.2%)	
Chemotherapy			0.555			0.523
Yes	24(88.9%)	170(84.6%)		56(87.5%)	138(84.1%)	
No	3(1.1%)	31(15.4%)		8(12.5%)	26(15.9%)	
Hormone therapy			0.001			<0.001
Yes	11(45.8%)	152(76.4%)		29(48.3%0	134(82.2%)	
No	13(54.2%)	47(23.6%)		31(51.7%)	29(17.8)	

LN, lymph node; HG, histologic grade; LVI, lymphovascular invasion; ER, estrogen receptor; PR, progesterone receptor; HER2, human epithelial growth factor receptor 2; EGFR, epithelial growth factor receptor.

**Table 2:** Multivariate cox regression analysis of DFS

		HR	95% CI	P-value
PD-L1 stromal immune	High vs. Low	0.084	0.011-0.645	0.017
Tumor size	T3&4 vs. T1&2	1.522	0.511-4.531	0.450
LN metastasis	Positive vs. Negative	1.429	0.360-4.331	0.726
LVI	Positive vs. Negative	4.574	1.305-16.036	0.018
Histologic grade	3 vs. 1&2	3.327	1.108-9.988	0.032
Progesterone receptor	Positive vs. Negative	0.414	0.133-1.289	0.128

LN, lymph node; LVI, lymphovascular invasion.
